# Sculpting the Intrinsic Modular Organization of Spontaneous Brain Activity by Art

**DOI:** 10.1371/journal.pone.0066761

**Published:** 2013-06-26

**Authors:** Chia-Shu Lin, Yong Liu, Wei-Yuan Huang, Chia-Feng Lu, Shin Teng, Tzong-Ching Ju, Yong He, Yu-Te Wu, Tianzi Jiang, Jen-Chuen Hsieh

**Affiliations:** 1 Department of Dentistry, School of Dentistry, National Yang-Ming University, Taipei, Taiwan; 2 LIAMA Center for Computational Medicine, National Laboratory of Pattern Recognition, Institute of Automation, Chinese Academy of Sciences, Beijing, China; 3 Integrated Brain Research Unit, Department of Medical Research and Education, Taipei Veterans General Hospital, Taipei, Taiwan; 4 Department of Biomedical Imaging and Radiological Sciences, National Yang-Ming University, Taipei, Taiwan; 5 Department of Music, School of Music, Taipei National University of the Arts, Taipei, Taiwan; 6 State Key Laboratory of Cognitive Neuroscience and Learning, Beijing Normal University, Beijing, China; 7 Institute of Brain Sciences, National Yang-Ming University, Taipei, Taiwan; 8 Key Laboratory for NeuroInformation of Ministry of Education, School of Life Science and Technology, University of Electronic Science and Technology of China, Chengdu, China; 9 Center for Neuropsychiatric Research, National Health Research Institutes, Taiwan; National Research & Technology Council, Argentina

## Abstract

Artistic training is a complex learning that requires the meticulous orchestration of sophisticated polysensory, motor, cognitive, and emotional elements of mental capacity to harvest an aesthetic creation. In this study, we investigated the architecture of the resting-state functional connectivity networks from professional painters, dancers and pianists. Using a graph-based network analysis, we focused on the art-related changes of modular organization and functional hubs in the resting-state functional connectivity network. We report that the brain architecture of artists consists of a hierarchical modular organization where art-unique and artistic form-specific brain states collectively mirror the mind states of virtuosos. We show that even in the resting state, this type of extraordinary and long-lasting training can macroscopically imprint a neural network system of spontaneous activity in which the related brain regions become functionally and topologically modularized in both domain-general and domain-specific manners. The attuned modularity reflects a resilient plasticity nurtured by long-term experience.

## Introduction

Michelangelo declared five centuries ago that “*a man paints with his brains and not with his hands.*” Artistic training is a form of complex learning that requires the learner to use experience to consolidate versatile perceptual, polysensory, skillful motor, complex cognitive and profound emotional elements [1]–[3]. Empathy and embodiment are critical constituents underpinning the holistic esthetic consciousness that enables an artist's work to resonate appraisal and appreciation [1], [2]. Painting is a form of creative expression in which drawing, composition, abstraction and other esthetics may serve to manifest the artist's expressive and conceptual intention through the perception and representation of intensity, color, tone, shape, texture, and rhythm. In other words, painting incorporates form, melody, coloration and energy into a sequence. Dancing is a performing art that embodies ideas, emotions and transformative thinking through a choreographic design of kinesthetic body actions that are typically rhythmically coordinated with music and conform to the movements of other dancers in space. Instrument performance (e.g., piano) consists of the use of music to produce an esthetic experience through stylistic and structured tones, harmonies, rhythms, forms, tempos, melodies, and dynamics. Instrument performance requires highly skilled ideomotor control, sight-reading, memorization, and improvisation. In addition to unique capacities specific to their virtuosity, all artists require heightened skills in computing kinematic information for producing movements with high accuracy and precision. Such a skill is mandated by the cerebellum, as part of the timing system for processing temporally organized events, engineering action-related information (velocity, intensity, and timing) automatically [4]. all artists require a heightened sensitivity for dynamic interplay between the self and the environment, esthetic appraisal, and a high level of sentience regarding the intention and feeling of others (i.e., the experience of embodiment and empathy) [1], [3] We hypothesized, in the context of learning-related brain neuroplasticity, that such diverse artistic-mind traits can be differentially echoed by their corresponding brain organizations, respectively, of which parallel information processing among the sets of related regions can be efficiently organized even in the resting state of the brain. The resting-state functional connectivity (rsFC) during low-frequency oscillations, as studied by functional magnetic resonance imaging (fMRI), may reflect the brain state of self-referential internal representation [5], exteroceptive and interoceptive deployment of attention [6], and readiness of the brain to engineer an instant mind operation [7]. rsFC can be rapidly sculpted by intensive short-term learning in a behaviorally specific manner coupled with contingent regional co-activations [8], [9]. Professional artists, however, are unique study subjects for human brain neuroplasticity, whose nurturing mandates long-term training which in turn may render his brain in the form of a network system, as represented by the constellations of the functional links among contingently associated regions. The functional dynamics of these consolidated neural ensembles, including modular processing, in brain networks can be better studied using topological approaches [10], [11].

In this study, we exploited graph theory-based network analysis [12] to investigate the architecture of brain connectivity networks from various artists (Table 1). The model of the rsFC network constructed based on the graph approach is composed of nodes (parcellated brain regions, see Table 2 for all neuroanatomical abbreviations) and edges (rs-connectivity between regions). The brain exhibits an efficient small-world modular architecture, characterized by a higher level of local connectedness but a very short average travel distance between nodes, with network communities interlinked by hub regions. Such a small-world structure is associated with high efficiency of parallel information processing [13]. At the overall topological level, the general network properties, as assessed by the degree of small-worldness [14], [15] and the network efficiency for parallel information processing [16], reflect the integrity of fundamental brain functions. At the modular level, the specific architecture reveals how the related brain regions coordinate activity among each other [12]. Furthermore, the centrality of an individual node reveals its role in inter- and intra-modular connections [17]. We studied the overall topological and modular features of artists (painters, dancers and pianists) and non-artist controls to determine artistic brain states that represent the unique mind traits for each art form. First, we probed how well intrinsic activity is coherently processed over the rsFC network in terms of small-worldness and network efficiency. Intuitively, these professional artists (especially dancers and pianists who are highly skillful performers) might show enhanced efficiency in the overall information processing within the rsFC network. Second, we hypothesized that there should be art-unique (shared among artists but not the controls) and artistic form-specific representations that resiliently mirror common and distinct traits, respectively, of the artistic mind. Notably, key regions subserving art-laden empathy-embodiment and esthetic appraisal [1], [18], such as the inferior frontal gyrus (IFG) and insula (INS), may show a distinct modular architecture in all artists' brain networks.

**Table 1:** Demographic data.

Group	Control	Painter	Dancer	Pianist
Gender (M/F)	12/15	12/16	13/17	12/17
Age				
Mean	24.5	23.0	**19.7	**21.8
SD	3.1	2.5	2.1	2.1
Max	30	33	23	26
Min	19	20	16	19
Duration of learning (years)				
Mean		11.3	11.2	**15.9
SD		4.1	3.8	2.4
Max		20	19	21
Min		4	2	12

** *P*<0.01; SD, standard deviation; Max/Min, maximal/minimal.

The number of participants did not differ between genders (Chi-square *P*>0.05). The dancer and pianist groups were significantly younger than the control group (two-tailed two-sample *t* test; dancer, *t*(55) = 6.94, *P*<0.001; pianists, *t*(54) = 3.84, *P*<0.001). The duration of learning differed among the three artist groups (one-way ANOVA, *F*(2,84) = 16.96, *P*<0.001). Post-hoc analysis using the Tukey HSD test revealed that the pianists had a longer duration of learning than the painters (*P*<0.001) and the dancers (*P*<0.001). There was no difference in the duration of learning between the painters and the dancers. The relatively younger age and preponderance of females in our artist sample group largely reflects the demographics of artist education in Taiwan. All the professional artists enrolled in this study were art students of art universities of Taiwan (mainly from Taipei National University of Arts). All students have been receiving special and dedicated programs of arts with stringent training and continuous education since primary school. More specifically, the program for the dancers is a unified system integrating high school and university education together.

**Table 2:** Abbreviations of neuroanatomical regions.

AAL Nomenclature				
		Regions		Regions
Cerebral	PreC	Precentral gyrus	ORBmid	Middle frontal gyrus (orbital part)
	ACG	Anterior cingulate and paracingulate gyri	ORBsup	Superior frontal gyrus (orbital part)
	AMYG	Amygdala	ORBsupmed	Superior frontal gyrus (medial orbital)
	ANG	Angular gyrus	PAL	Lenticular nucleus, pallidum
	CAL	Calcarine fissure and surrounding cortex	PCL	Paracentral lobule
	CAU	Caudate nucleus	PCG	Posterior cingulate gyrus
	CUN	Cuneus	PCUN	Precuneus
	DCG	Median cingulate and paracingulate gyri	PHG	Parahippocampal gyrus
	FFG	Fusiform gyrus	PostC	Postcentral gyrus
	HES	Heschl gyrus	PUT	Lenticular nucleus, Putamen
	HIP	Hippocampus	REC	Rectus gyrus
	IFGoper	Inferior frontal gyrus (opercular part)	ROL	Rolandic operculum
	IFGtriang	Inferior frontal gyrus (triangular part)	SFGdor	Superior frontal gyrus (dorsolateral)
	INS	Insula	SFGmed	Superior frontal gyrus (medial)
	IOG	Inferior occipital gyrus	SMA	Supplementary motor area
	IPL	Inferior parietal, but supramarginal and angular gyri	SMG	Supramarginal gyrus
	ITG	Inferior temporal gyrus	SOG	Superior occipital gyrus
	LING	Lingual gyrus	SPG	Superior parietal gyrus
	MFG	Middle frontal gyrus	STG	Superior temporal gyrus
	MOG	Middle occipital gyrus	THA	Thalamus
	MTG	Middle temporal gyrus	TPOmid	Temporal pole: middle temporal gyrus
	OLF	Olfactory cortex	TPOsup	Temporal pole: superior temporal gyrus
	ORBinf	Inferior frontal gyrus (orbital part)		
Cerebellar	Crus 1	Crus cerebelli I	Vr 1/2	Vermis lobule I/II
	Crus 2	Crus cerebelli II	Vr 3	Vermis lobule III
	CL 3	Cerebellar lobule III	Vr 4/5	Vermis lobule IV/V
	CL 4/5	Cerebellar lobule IV/V	Vr 6	Vermis lobule VI
	CL 6	Cerebellar lobule VI	Vr 7	Vermis lobule VII
	CL 7b	Cerebellar lobule VIIB	Vr 8	Vermis lobule VIII
	CL 8	Cerebellar lobule VIII	Vr 9	Vermis lobule IX
	CL 9	Cerebellar lobule IX	Vr 10	Vermis lobule X
	CL 10	Cerebellar lobule X		

Other abbreviations used in the main text	
AAL	Automated Anatomical Labeling
FDR	False Discovery Rate
IFG	Inferior Frontal Gyrus
MNS	Mirror Neuron System
mOFC	Medial Orbitofrontal Cortex
rsFC	Resting-state Functional Connectivity

## Materials and Methods

### Participants

Three groups of different artistic professions (painters, dancers and pianists, n = 30 for each group) and one group of non-artists (control group, n = 30) were recruited for the study (see Table 1 for detailed demographic data). All participants were right-handed, and the artists were all recruited from professional music colleges or universities of arts. Six participants were excluded in the subsequent analysis due to interference caused by head movement during scanning (see below). The study protocol was approved by the Institutional Review Board of Taipei Veterans General Hospital, and written informed consent was obtained from all participants.

### Image acquisition and preprocessing

Resting-state functional MRI images (rs-fMRI) were acquired on a 3T MAGNETOM Trio™ (Siemens, Erlangen, Germany) of National Yang-Ming University of Taiwan, using echo-planar imaging (EPI), with the following parameters: repetition time (TR) = 2500 ms, echo time (TE) = 30 ms, 40 axial slices with slice thickness = 3.4 mm, flip angle = 90°, field of view (FOV) = 220×220 mm^2^, matrix size = 64×64, and voxel size = 3.4 mm×3.4 mm×3.4 mm. The duration of EPI scan was 510 sec, consisted of 204 volumes. All subjects were scanned with their eyes closed in a supine position. Structural 3D T1-weighted images for each subject were acquired using a rapid acquisition, gradient echo (MPRAGE) sequence with the following parameters: TR = 2530 ms, TE = 3.03 ms, flip angle = 7°, FOV = 224×256×192 mm^3^). The first four volumes were discarded due to the magnetic saturation effect. Preprocessing was performed using statistical parametric mapping (SPM8, http://www.fil.ion.ucl.ac.uk/spm) with the following steps: the correction of slice timing, the realignment for head movement correction (6-parameter rigid body transformation), the spatial normalization to the T1 template image, and spatial smoothing with an 8 mm FWHM Gaussian kernel. Participants with significant head movement (translation >2 mm or rotation >2° in any volume) during scanning were excluded. For the time series of each voxel of the normalized image, linear regression was performed to remove the effects of (a) head motion (using the six motion parameters estimated from rigid-body realignment), (b) the signals from white matter regions, (c) the signals from CSF regions, and (d) detrended with the shift of BOLD signals. The pre-processed images were used for the following steps of network construction. We did not perform global signal regression due to the recent debate on the potential distortion on correlation patterns [19], [20]. The resulting time series was band-pass filtered (0.01 Hz∼0.1 Hz) to extract the low-frequency oscillating components that contributed to rsFC.

### Construction of connectivity network

#### Definition of node

The node of a network was defined as the parcellated brain regions. Parcellation was performed according to Automated Anatomic Labeling (AAL) atlas, which consists of 90 cortical and 26 cerebellar anatomical areas (see Table 2 for the anatomical regions and abbreviations). The resulting parcellated templates were then respectively overlapped with the pre-processed functional images data. For each node, the mean time series was calculated by averaging the time series of each voxel in that node.

#### Definition of edge

The edge of a network was defined as the degree of correlation between the mean time series of each pair of nodes. Pearson correlation was performed for each pairwise time series. The resulted N-by-N ‘r-value matrix’ was then converted to a ‘z-score matrix’ using Fisher's r-to-z transformation to obtain a better normality of the correlation. Finally, the z-score matrix was rectified to obtain the absolute value, as the weight of edge. We then thresholded the z-score matrix at different levels of sparsity, using minimum spanning tree (MST) method, followed by global thresholding [21]. In brief, for each node, we selected the edge of the highest weight to keep the node contacted with at least on neighbor. The local thresholding step was iterated until all nodes are able to contact with one another, forming a *backbone* in which no island exists. Secondly, we added in the edges with the strongest weight, ranked by all the edges, into the backbone network. The ‘growing’ step was iterated until the number of edges meets with the assigned sparsity (i.e. global threshold). In order to investigate the effect of different network sparsity, networks were constructed with cost = 0.03 to 0.40, with a 0.01 increment. For a weight network, the edge weight over the threshold was kept, and for a binary network, the edge weight over the threshold was set to one.

### Statistical analysis

#### Analyses of the topological metrics of brain architecture

Clustering coefficient (*C_p_*), characteristic path length (*L_p_*) [15], global efficiency (*E_global_*), and local efficiency (*E_local_*) [22] were assessed (see Text S1 for full description on comparison in the topological metrics).

#### Difference in the modular architecture between groups

We investigated the difference in (a) modularity, (b) the number of partitioned modules, and (c) similarity between two modular partitions. The similarity between two partitions was quantified by their normalized mutual information (NMI) [23]. Between each of the artist group and the control group, modularity and the number of modules were compared, using independent two-sample *t* test. Similarity was compared by a permutation procedure. Briefly, we calculated the averaged pairwise NMI across all participants within a group. We tested the hypothesis that the averaged within group pairwise similarity is higher than the averaged between group pairwise similarity, which indicates a genuine difference in similarity between groups [23], [24]. The permutation procedure was performed by varying the group membership, with 10000 permutations.

For the analyses of the topological metrics, modularity and modular architecture, all between-group comparison was performed on the binarized networks across a range of network cost (0.03∼0.40). For the analyses of group level modular architecture and modular assignment of nodes (see below), we analyzed the network with cost = 0.03, which preserved the strongest inter-regional rsFC.

#### Visualization of Difference in the modular architecture at the group level

At the group level, to visualize the difference in modular architecture between groups, we performed the procedure according to [23]: First, within a group, we selected the participant which modular architecture shows the highest similarity with the other participants, as a ‘representative network’. Secondly, all the other participants' modular architecture was matched to this representative network. Finally, each node was labelled by the most frequently occurring label among the participants within the group. The frequency quantified the confidence of the assignment of nodes.

#### Difference in the modular assignment of specific nodes

To directly test the difference in modular assignment of a specific node of interest (NOI), we computed the similarity of module labels of two subjects, in terms of the functional community of the NOI. For a given NOI, to each subject, we labelled all the other nodes ‘1’ if they shared the same module as the NOI and ‘0’ if not. Similarity of module labels was calculated as Pearson's phi, the Pearson correlation for dichotomous variable, between each pair of subjects. For the NOI, a higher phi value indicates a higher similarity of modular architecture between subjects. We tested the hypothesis that the averaged within group pairwise similarity is higher than the averaged between group pairwise similarity, which indicates a genuine difference in similarity between groups [23]. The permutation procedure was performed by varying the group membership, with 10000 permutations. Because the tests were performed for 116 NOIs, we adjusted the alpha value for significance using Bonferroni corrections (adjusted alpha = 0.05/116).

#### Robustness to methodological variations

We analyzed the between-group difference in modularity and modular architecture of a weighted network derived from the rsFC data, using the same analysis procedure stated above.

## Results

### Topological characteristics of the rsFC networks (Figure 1)

**Figure 1:**
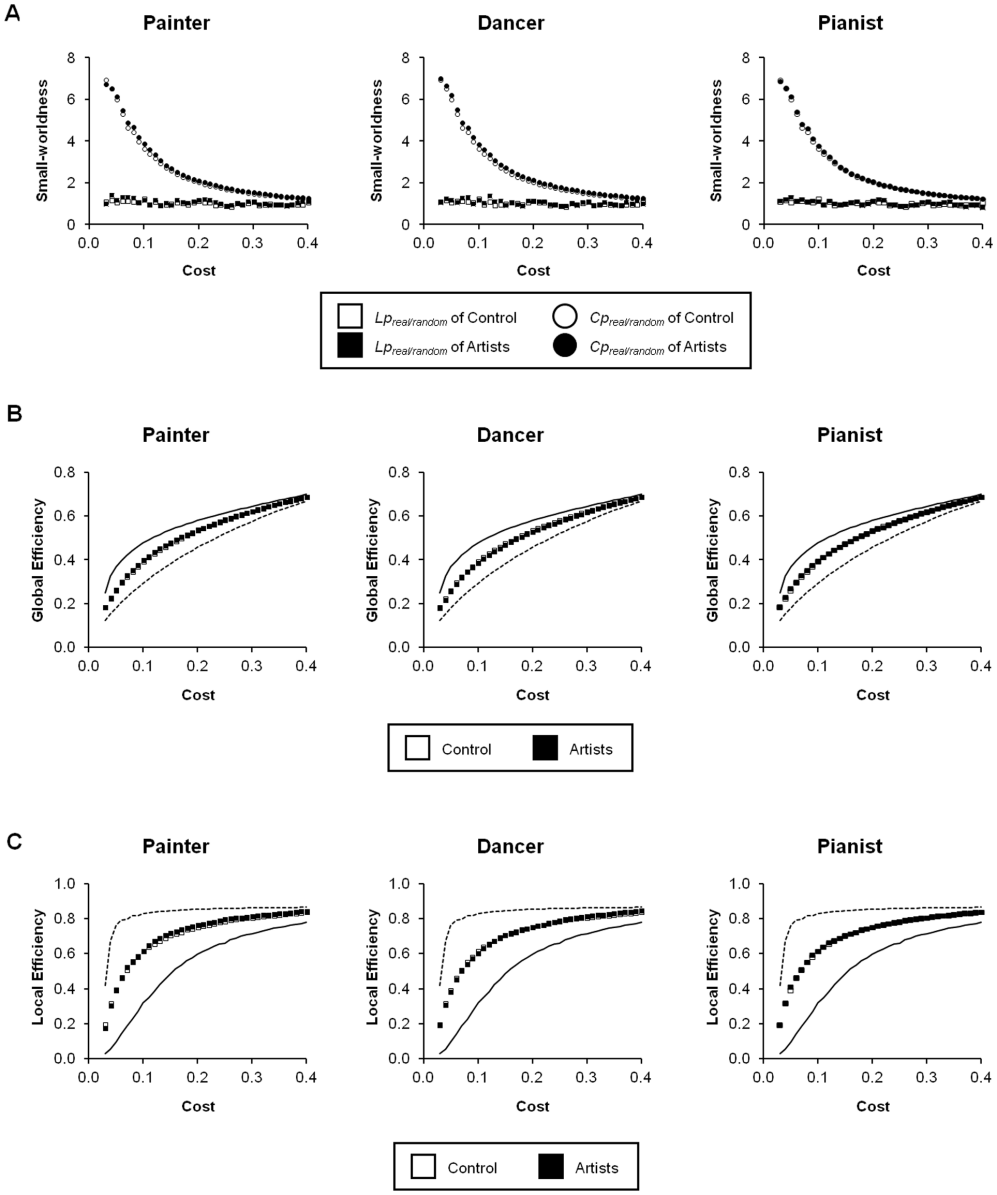
The topological metrics. The topological metrics related to (**A**) Small-worldness (**B**) global efficiency and (**C**) local efficiency of the respective artist groups (black) are plotted over the range of costs and overlay the results from the control group (white). The dashed lines and the continuous lines denote the efficiencies of the theoretical regular and random networks, respectively, as derived from the real network. No significant difference was found for the metrics between the artist and control groups.

We compared various metrics (clustering coefficient, *C_p_*; characteristic path length, *L_p_*; global efficiency, *E_global_*; and local efficiency, *E_local_*) as a function of cost among real, random and regular networks between all groups. Small-world characteristics of brain organization, in terms of the rsFC networks, were affirmed in all groups. We did not find significant differences in the topological metrics across groups (Figure 1). This result implies that artistic training does not alter the general efficiency of the brain network in terms of short- and long-range connectedness and communication efficiency.

### Difference in modularity and modular architecture between groups (Figure 2)

**Figure 2:**
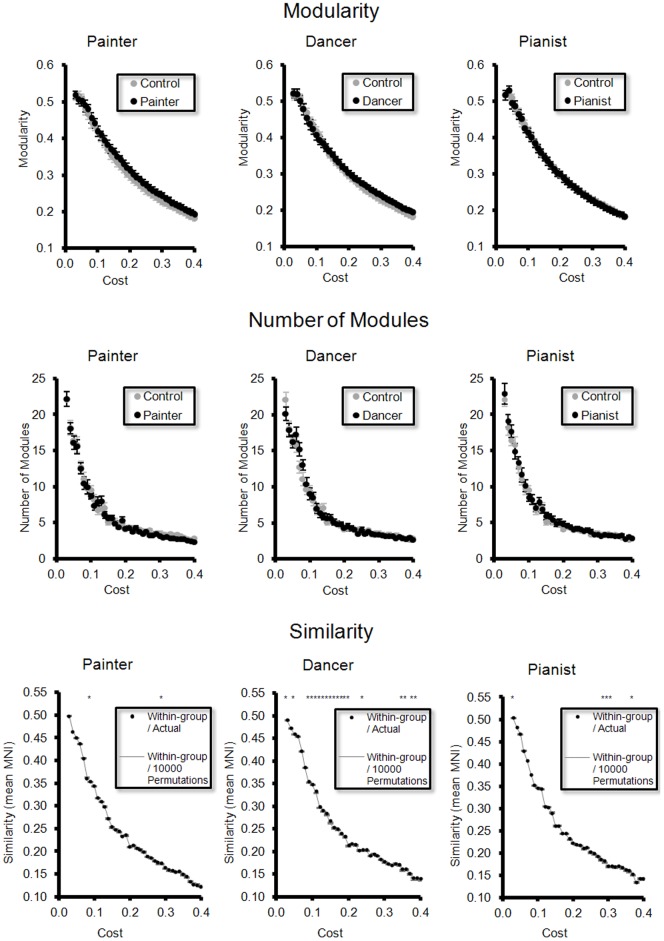
Difference in modularity and modular architecture. There is no significant difference in (**A**) modularity and (**B**) the number of modules of the rsFC networks between each artist group and the control group, over the range of costs. For (**C**) modular architecture, there is significant difference in similarity (assessed by NMI) predominantly between the dancer group and the control group (asterisks). The bar denotes standard error of means.

In all the three artist groups vs. the control group, we did not find significant difference in modularity (Figure 2A) and the number of modules (Figure 2B), for the full range of network costs. In painter vs. control and pianist vs. control, we did not find significant difference in similarity of modular architecture. However, in dancer vs. control, we found significantly difference in similarity, at different network costs (Figure 2C).

### Visualization of difference in the modular architecture at the group level (Figure 3–4)

**Figure 3:**
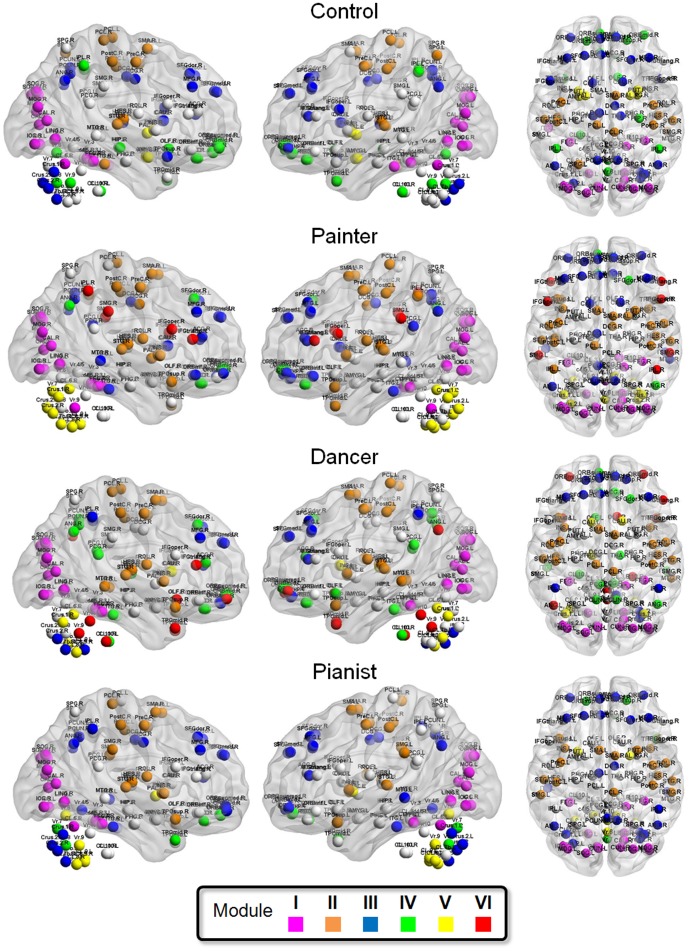
Visualization of the representative modular architecture at the group level. In each group, the participant which modular architecture shows the highest similarity with the other participants was selected. All the other participants' networks were matched to the participant by maximizing the overlap between all subjects. The color labels were assigned to each node according to as the most frequent label across all of the subjects. For the display purpose, only the major modules, defined as the modules consisting of at least six nodes, are shown.

We investigated the major module, defined as the modules consisting of at least six nodes (Figure 3). In the control group, the cortical nodes were decomposed into five major modules Module I consists of the nodes relevant to visual processing, including the bilateral IOG, MOG, SOG, CAL, CUN, LING, and FFG (see Table 2 for all neuroanatomical abbreviations). Module II consists of the nodes relevant to sensorimotor functions, including the sensory subnetwork (the bilateral PostC and PCL, and the left SPG), the motor subnetwork (the bilateral PreC and the right SMA), and the auditory subnetwork (the bilateral HES and STG). Module III consists of the nodes related to the default mode network (the bilateral SFGmed, the left ACG, the left MFGorb, and the bilateral PCUN and ANG), the frontal control subnetwork (the bilateral SFGdor and MFG) and part of the subcortical motor subnetwork (the bilateral CAU and THA). Module IV consists of the nodes relevant emotional processing, including the bilateral SFGorb, SFGsupmed and REC. Finally, Module V consists of part of the subcortical motor network (the bilateral PAL and PUT) and the bilateral AMYG. The findings are consistent with the modular architecture previously reported [10], [11]. The cerebellar nodes were principally decomposed into two major modules (Module III and Module V). Notably, the Crus I and II and the DMN were assigned to the same module (Module III), consistent with previous findings from ICA [25] and rsFC analysis [26].

In the artist groups, we found an additional major module in the dancer and the painter groups. In the painter group, the inferior frontal gyrus (including the bilateral IFGoper and IFGtriang) and the bilateral SMG and the right IPL form a module. In the dancer group, the bilateral MFGorb and TPOmid, the right IFGtriang, and the cerebellar nodes, form a module. The degree of change in modular architecture is consistent with the difference of similarity between the dancer and the painter groups (Figure 2C).

**Figure 4:**
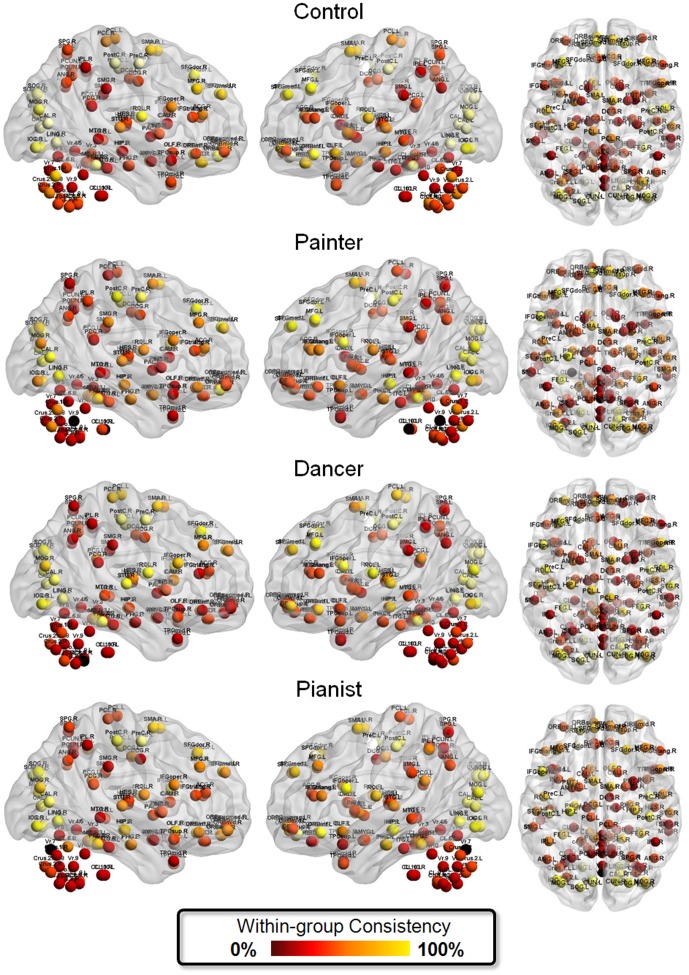
The within-group consistency of modular architecture. In each group, the consistency of assigning a node to a specific module was quantified as the frequency that the node belongs to the module, across all participants. The greater consistency means the higher confidence of module assignment of the node.

Across all groups, we found high inter-subject consistency of modular architecture at the occipital module (Module I) and the sensorimotor module (Module II), consistent with previous findings [23]. In contrast, the nodes within the posterior parietal lobe, the temporal lobe and the cerebellum showed lower inter-subject consistency of modular architecture (Figure 4).

### Difference in the modular assignment of specific nodes (Figure 5)

**Figure 5:**
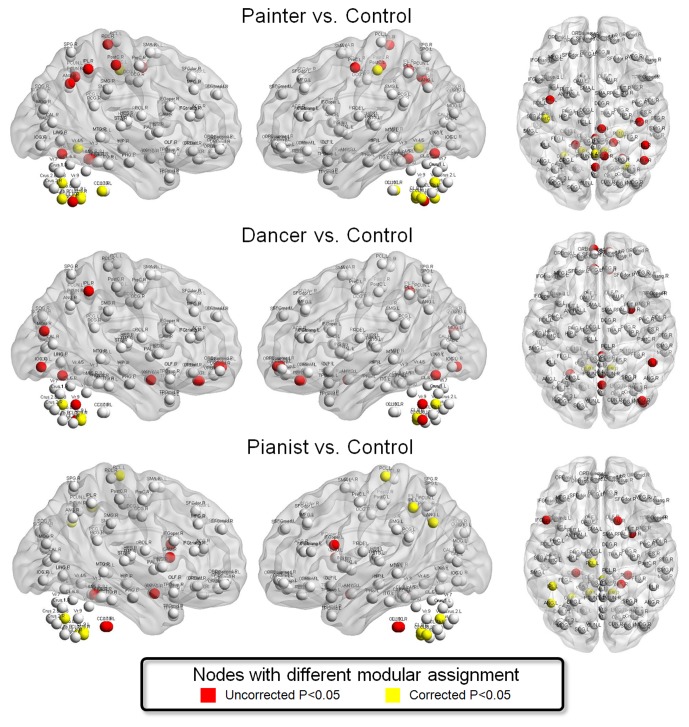
Difference in the modular assignment of specific nodes. Between each artist group and the control group, the nodes with significantly different modular assignment were displayed. The nodes with significant difference with and without correction for multiple comparisons were labeled as yellow and red, respectively.

In painter vs. control, the regions with significantly different functional communities were found at the left PostC and the bilateral cerebellum (including the right CL_7b, the left CL_8, the bilateral CL_9, the right CL_10, Vr_4/5, and Vr_8) (P<0.05 adjusted for Bonferroni correction). Sub-significant changes were found at the left PreC, the right PostC, the right IPL, the right ANG, the right PCUN, the right PCL, the bilateral CL_4/5, , the right CL_8, , Vr_1/2, and Vr_6 (P<0.05, uncorrected) (Figure 5).

In dancer vs. control, the regions with significantly different functional communities were found exclusively at the bilateral cerebellum (including the bilateral CL_9 and Vr_8) (P<0.05 adjusted for Bonferroni correction). Sub-significant changes were found at the bilateral ORBsupmed and REC, the right AMYG, the right MOG, the right IOG, the right IPL, the left CL_8, the Vr_1/2, the Vr_6, and the Vr_9 (P<0.05, uncorrected) (Figure 5).

In pianist vs. control, the regions with significantly different functional communities were found at the left parietal cortex (including the left IPL, the left ANG, the left PCL) and the bilateral cerebellum (including the left CL_8, the bilateral CL_9, the Vr_1/2, and Vr_8) (P<0.05 adjusted for Bonferroni correction). Sub-significant changes were found at left IFGoper, the right AMYG, the right CAU, the right CL_4/5, and the bilateral CL_10 (P<0.05, uncorrected) (Figure 5).

### Robustness to methodological variations (Figure 6)

**Figure 6:**
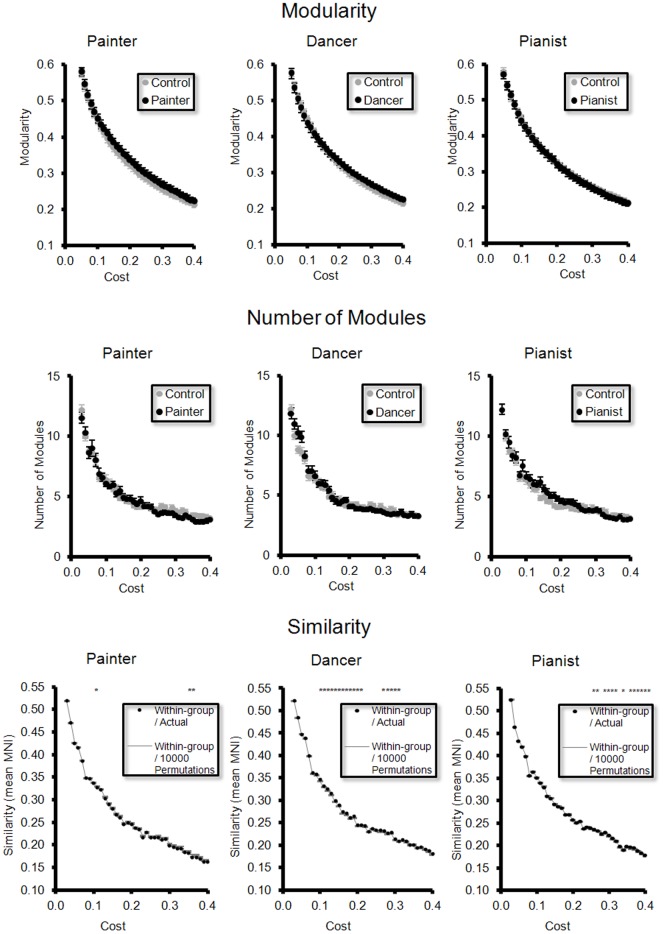
Robustness to methodological variations. For the weighted network, there is no significant difference in (**A**) modularity and (**B**) the number of modules of the rsFC networks between each artist group and the control group, over the range of costs. For (**C**) modular architecture, there is significant difference in similarity (assessed by NMI) predominantly between the dancer group and the control group (asterisks). The bar denotes standard error of means.

For the weighted rsFC network, we did not find significant difference in modularity and the number of modules between each artist group and the control group. We found significant difference in modular architecture (assessed by similarity) between the dancer group and the control group (Figure 6). The findings are consistent with the findings derived from the binary network (Figure 2C).

## Discussion

### The similar efficiency of the rsFC network between artists and control groups

Economical organization of a complex network is critical for efficient information processing and optimal functioning [13]. We affirmed that artists' brains, regardless of the artistic profession, exhibit small-world characteristics. While decreased efficiency is often associated with a disrupted network related to brain disease [27], [28], higher efficiency of the rsFC network has been reported in people with a higher intelligence quotient (IQ) based on the level of verbal and non-verbal knowledge and reasoning [29]. One might intuitively expect that an artist, given the capacity of creating novel aesthetic experiences, may show increased network efficiency. However, the absence of an efficiency difference between artist and control groups strongly suggests that the artistic brain is distinct from the intellectual brain in the context of differential architecture of the connectivity network, which is representative of the discordant essence of mind operations. As opposed to the “tough-minded” intellectual performance that is associated with more logic, objective, formal, and conventional features, the “tender-minded” artistic excellence can be associated with more intuitive, subjective, emotional, and individualistic dispositions [30]. Thus, it is plausible that the artistic mind, as opposed to the intellectual mind, is driven by the intricate topological rewiring of networks without essential alterations in the overall efficiency of the brain.

### The art-unique organization of modular architecture

Our findings revealed that the cerebellar regions showed changing functional communities, consistently in each of the artist groups, compared to the control group (Figure 4). The cerebellum, which is part of the timing system for processing temporally organized events, computes kinematic information for producing movements with high accuracy and precision [4]. The cerebellum may engineer action-related information (velocity, intensity, and timing) automatically [4]. These differential connection patterns of engagement of cerebellar nodes in different artists, disclosed by divergent modular organizations, echo the artists' distinct action profiles. The art-unique organization in the cerebellum may pinpoint the high-functioning status of the “internal-model control systems” for timed voluntary movement control during complex action and associated mental effort [31] in all artists.

### The artistic form-specific neuroplasticity of modular architecture

We detected artistic form-specific resilience of brain organization that echoes the versatile nature of different arts. A professional painter creatively forms a visual Gestalt that embraces the creator's own intention through the intensity, color, shape and texture of the materials [32]. A professional dancer actively relates his or her body language with the present through fine control of movements in sequence for a body schema under choreographic design and simultaneously negotiates kinesthetic body actions (with others in the case of group dancing) usually synchronized to music [33]. A professional pianist masters both ideomotor control, e.g., keyboard-playing, and perceptual skills, e.g., sight-reading and complex auditory comprehension, in the form of syntactic language to elaborate the perceptual and expressive meaning of music [34].

#### Painters

The parietal sensorimotor network, including the left PreC, the bilateral PostC and the right PCL, showed significant changes in modular assignment (Figure 5). In contrast to the control group, in the painters, these nodes formed a greater module, which consisted of the bilateral ROL, SMA, and INS (Figure 3). Such an extension functional community suggests an extended integration of perception and motor control in the painters. The bilateral INS is a critical region for interoception and emotion, representing the feeling of “the sentient self” [35]. The link between the sensorimotor nodes with the INS suggests the visceral perception and emotion as a critical part of esthetic processing [18].

#### Dancers

The mOFC nodes (including the bilateral ORBsupmed and REC) are critical to reward processing and esthetic evaluation [18]. They encode the heightened esthetic value across polysensory (visual, auditory, olfactory and gustatory) modalities and serves as a core of the esthetic network [18].

#### Pianists

The IFGoper_L, as part of the Broca's area, is associated with the processing of information with syntactic structure, including musical phrases and complex actions [34], [36]. The left ANG is critical for binding multimodal sensory information for integral cross-modal learning [37] and musical semantic memory [38].

### Further considerations

Embodied simulation-driven empathic feelings are critical and emphasized in artistic training. These feelings represent consciousness and expressive actions that create a dynamic interplay between the self and other people (artistic gesture) or the environment. The IFG, IPL, and STG are key structures of the core human mirror neuron system (MNS) [39]. These critical neural correlates resonate with the empathic mind and artistic gesture through recapitulation of kinesthetic perception and action in a reciprocal way, and may show an art-unique change in modular architecture. We here did not find significantly different modular assignment in the MNS-related nodes. The negative finding may not dispute the importance of the MNS in the artists, but could reflect the effect from the following factors.

#### Sample size

The importance of the sample size in rsFC research has been recently highlighted [40]. Because the brain neuroplasticity is a very subtle effect, a larger sample size would be necessary to reveal the reliable changes in modular architecture.

#### Gender and age factors

Both factors could influence the rsFC networks [11], [41]. In the current study, the participants in the artist group were younger and more females than males. The relatively younger age and preponderance of females in our artist sample group largely reflects the demographics of artist education in Taiwan.

#### The individual variations in duration of learning

All the professional artists enrolled in this study were art students of art universities of Taiwan (mainly from Taipei National University of Arts). All students have been receiving special and dedicated programs of arts with stringent training and continuous education since primary school. However, the time when the artists started to learn the professions may vary. The heterogeneity in duration of learning may influence brain neuroplasticity.

#### The individual variations in the way of artist training

Because artistic training is heavily relying on tutoring, the development of artistry may vary across individual artists.

In the future study regarding art-related neuroplasticity, we aim to increase the sample size, with a stringent control for the gender and age of the artists, and minimize the individual variations in experience about learning art.

## Conclusions

### The neuroplasticity of complex learning is imprinted in the topology of neural networks in the brain

Learning-related plasticity is associated with resilient changes in neural connectivity at multiple organizational levels. At the microscopic level, learning is associated with spike-timing-dependent plasticity [42] and a plasticity of synaptic efficiency based on contingency [43]. At the macroscopic level, learning-related plasticity is associated with the experience-driven consolidation of inter-regional functional links as a form of “system memory” that dynamically recapitulates intensive training [8]. Our study demonstrates the flexibility and adaptability of brain networks involved in complex learning, as revealed by the protean modular organization of the brain [44]. In contrast to previous studies on short-term simple perceptual and motor training [8], [9], attaining artistic virtuosity is a process of *complex learning* that mandates long-term training to achieve meticulous orchestration of sophisticated polysensory, motor, cognitive and emotional elements of mental capacity to holistically harvest an aesthetic creation. For the first time in the literature, we show that this type of extraordinary and outlasting training can macroscopically imprint a neural network system of spontaneous activity even in the resting state that results in brain regions becoming functionally and topologically modularized in both domain-general and domain-specific manners. The artists' brain features economical properties and can achieve a high level of efficiency of both local information processing and global communication between different regions without incurring any additional cost in the default state of the average brain. Using a graph-theory-based approach, we report that artists' brains are architected in a hierarchical small-world organization where art-unique and artistic form-specific brain states collectively mirror the mind states of artistic virtuosos. The dynamic plasticity of modular organization confers the evolvability of the human brain. Our innovative work has substantially advanced the understanding of the brain resilient plasticity, consolidation of neural network after long term complex learning, and brain-mind correspondence. Our work not only have profoundly furthered the mechanistic understanding of neuroaesthetics and the brain architectures of artistic professions but also have potential implications on how an injured brain can resume functioning connections through intensive and sustained rehabilitation in general and art therapy in specific.

## Supporting Information

Text S1 **Graph-based network analysis.**
(DOCX)
