# Correction: Sculpting the Intrinsic Modular Organization of Spontaneous Brain Activity by Art

**DOI:** 10.1371/annotation/da5a8473-6c62-4921-aea7-539175b1a9fa

**Published:** 2013-07-09

**Authors:** Chia-Shu Lin, Yong Liu, Wei-Yuan Huang, Chia-Feng Lu, Shin Teng, Tzong-Ching Ju, Yong He, Yu-Te Wu, Tianzi Jiang, Jen-Chuen Hsieh

An affiliation for the first author is missing. Chia-Shu Lin is affiliated with institution 1, Department of Dentistry, School of Dentistry, National Yang-Ming University, Taipei, Taiwan, but is also affiliated with institution 3, Integrated Brain Research Unit, Department of Medical Research and Education, Taipei Veterans General Hospital, Taipei, Taiwan. 

In addition, the order of affiliations of the last author is incorrect. Jen-Chuen Hsieh is affiliated first with institution 7, Institute of Brain Sciences, National Yang-Ming University, Taipei, Taiwan, then 3, Integrated Brain Research Unit, Department of Medical Research and Education, Taipei Veterans General Hospital, Taipei, Taiwan, and finally 9, Center for Neuropsychiatric Research, National Health Research Institutes, Taiwan. In addition, affiliation institution 7 was given incorrectly. The correct institution is: 2, Institute of Brain Science, National Yang-Ming University, Taipei, Taiwan.

