# Supplementary material for: Sculpting the Intrinsic Modular Organization of Spontaneous Brain Activity by Art
Source: PLoS One. 2013 Jun 26;8(6):e66761. doi: 10.1371/journal.pone.0066761 (PMC3694132; doi:10.1371/journal.pone.0066761)
Supplement: Text S1 — Graph-based network analysis. (DOCX) [file pone.0066761.s001.docx]

**Text S1: Graph-based Network Analysis**

*Analyses of the Topological Metrics of Brain.* Comparison in the topological metrics across groups was performed in the following steps:

1. *Calculation of the topological metrics.*
   1. For any node *i* in a graph *G* composed of *n* nodes, *C_p_* and *L_p_* were obtained by averaging the measures of each node, across all nodes in the graph:

$$Lp=\frac{1}{n}\sum_{i\in G} Lp_{(i)}=\frac{1}{n}\sum_{i\in G} \frac{\sum_{i\neq j\in G}d_{ij}}{n-1}$$

*t_i_* is the number of triangles including node *i*, *k_i_* is the number of edges connected to node *i*, and *d_ij_* is the shortest path length between node *i* and the other node *j* of the network. γ is the ratio of the *C_p_* of the real network to that of the random network (γ = *Cp_real* /*Cp_random*). λ is the ratio of the *L_p_* of the real network to that of the random network (λ = *Lp_real* /*Lp_random*). The small-world organization is characterized by a higher clustering coefficient (i.e., γ >1) and a similar average shortest path length (λ ≈ 1) [1].

- 1. *E_global_* and *E_local_* was calculated as:

$$E_{global}=\frac{1}{n}\sum_{i\in G} \frac{\sum_{i\neq j\in G} d_{ij}^{-1}}{n-1}$$

$$E_{local}=\frac{1}{n}\sum_{i\in|G} E_{local(i)}=\frac{1}{n}\frac{1}{n_{G_{i}}(n_{G_{i}}-1)}\sum_{j\neq k\in G} \frac{1}{d_{jk}}$$

Here $n_{G_{i}}$denotes the number of nodes within a subgraph *G_i_*, consisting of all neighbors of node i. Note that *E_local_* were obtained by averaging the *E_local_* across all nodes in the graph [2].

1. *Constructing reference networks.* The reference networks include a *random* *network* of randomized structure and a *regular* *network* of lattice structure that share the size and the degree distribution (the probability distribution of the degree, i.e., the number of connections of each node, over the whole network) of the original *real* *network* of the respective group. Both *reference networks* were generated by re-wiring the original *real network* with 100 iterations using Brain Connectivity Toolbox (https://sites.google.com/a/brain-connectivity-toolbox.net/bct).
2. *Comparing topological metrics between groups.* The topological metrics ( *E_global_*, *E_local_*, *γ* and *λ*) were calculated as a function of cost (*K*), defined as:

$$K=\frac{1}{n(n-1)}\sum_{i\in G} k_{i}$$

*k_i_* is the number of edges of node *i*, and *n* is the total number of the nodes in the graph *G*. The cost is the proportion of the existing number of edges to that of all possible edges of the network [3]. A two-sample *t* test was conducted separately on each of the metrics between the respective artist group and the control group over a range of *candidate* costs (ranging from *K* = 0.03 to *K* = 0.4 with an increment 0.01). Correction for multiple comparisons over the candidate costs was performed using the Bonferroni approach.

*Optimizing Modularity*

Modularity analyses (Figure 1) were then performed on these non-directional and un-weighted networks (each edge was binarized to either 1 or 0). The extent to which the network is partitioned into non-overlapping groups (modules) was quantified as modularity (*Q*), using the spectral algorithm [4] as the following:

$$Q(p)=\sum_{s=1}^{N_{M}} \left[\frac{l_{s}}{L}-\left(\frac{d_{s}}{2L}\right)^{2}\right]$$

*N_M_* is the number of modules partitioned from the network. *L* is the number of total edges in the network. *l_s_* is the number of edges within a module *s. d_s_* is the sum of the degrees of the node in a module *s*. The algorithm computed the specific partition *p* of the network by maximizing *Q*. The topology of the network was then visualized with the BrainNet Viewer (http://www.nitrc.org/projects/bnv/). Note that the algorithm is non-deterministic, i.e., the resulting Q would vary between different runs of calculation. To obtain a modular configuration with the highest degree of modularity, we performed modularity optimization for 100 times, and reported the resulting partition p with the modular organization with the highest Q.

References:

1. Humphries MD, Gurney K (2008) Network 'small-world-ness': a quantitative method for determining canonical network equivalence. PLoS One 3: e0002051.

2. Latora V, Marchiori M (2001) Efficient behavior of small-world networks. Phys Rev Lett 87: 198701.

3. Latora V, Marchiori M (2003) Economic small-world behavior in weighted networks. The European Physical Journal B 32: 15.

4. Newman ME (2006) Modularity and community structure in networks. Proc Natl Acad Sci U S A 103: 6.
